# Novel Study Antimicrobial and Biocompatibility Effect of Magnesium Silver Alloys 1% on Bovine Bacterial Species

**DOI:** 10.1155/2023/8627515

**Published:** 2023-03-27

**Authors:** Yousra Nomier, Jessica Meiβner, Manfred Kietzmann

**Affiliations:** ^1^Department of Pharmacology and Toxicology, Pharmacy College, Jazan University, Jazan, Saudi Arabia; ^2^Department of Pharmacology, Toxicology and Pharmacy, University of Veterinary Medicine Foundation, Hannover, Germany

## Abstract

MgAg 1% alloys inhibit bacterial growth during the dry-off period. MgAg1% silver and magnesium amount was determined and the supernatant was used for the detection of antibacterial tests against *S. aureus* and *E. coli* and *Geobacillus stearothermophilus* var. *calidolacits*. The antibacterial effect of AgNO_3_ solution and degradation medium of MgAg1% sticks were evaluated. The bouillon dilution test showed a 5-fold reduction in bacterial colonies. Minimum inhibitory concentration (MIC) and minimum bactericidal concentration (MBC) calculations were used to test the antibacterial activity. The Brilliant Black Reduction Test (BRT-MRL screening test) showed inhibition of *Geobacillus stearothermophilus* bacteria at AgNO_3_ concentrations 0.01 mmol/l and 3 ml of degradation medium. Results were satisfying, concerning biocompatibility, degradation, and antibacterial effects.

## 1. Introduction

Mastitis is the inflammation of the mammary glands, which occur primarily due to intramammary bacterial infection [1]. Bovine mastitis remains the largest hazard in the global dairy industry since it causes major economic loss due to the reduction in milk yield. Most cases of subclinical mastitis are caused by contagious mastitis bacteria such as *Staphylococcus aureus (S. aureus)* and *Streptococcus agalactiae (Str. agalactiae)*, whereas other environmental pathogens like *Str. uberis* and *Escherichia coli (E. coli)* are also considered to cause chronic mastitis [2]. Local antibiotic treatments of the mammary glands are the most common treatment method for mastitis [3]. However, sometimes antibiotics show poor responses and these differ for different pathogens causing mastitis [4]. Each usage of antibiotics causes the risk to develop antibiotic resistance in pathogenic and commensal bacteria. Therefore, alternatives to antibiotic compounds have to be investigated. A biodegradable magnesium silver alloy (MgAg 1%) could be a promising tool to treat bovine mastitis. Magnesium (Mg^2+^) is an attractive material for biodegradable implants because of its low thrombogenicity, well-known biocompatibility, and lightweight [5]. The *in vivo* corrosion of Mg^2+^-based implants involves the formation of soluble nontoxic oxide which is excreted via the urine [6]. Silver (Ag^+^) is a broad-spectrum antibiotic with antiseptic and anti-inflammatory properties that are not yet associated with bacterial resistance [7]. Also, its ability to immediately destroy microorganisms by disturbing the bacterial cell membrane function and stopping cellular respiration recognizes it as an important element [8]. It is considered effective at low concentrations in addition to its therapeutic activity [9]. This study aimed to investigate the ability of MgAg1% alloy to inhibit bacterial growth during the dry-off period. We hypothesize that MgAg1% alloy is effective in controlling bacterial growth during the dry-off period.

## 2. Materials and Methods

### 2.1. Preparation of Magnesium-Silver 1% (MgAg1%) Stick and Silver Nitrate

**Figure 1:**
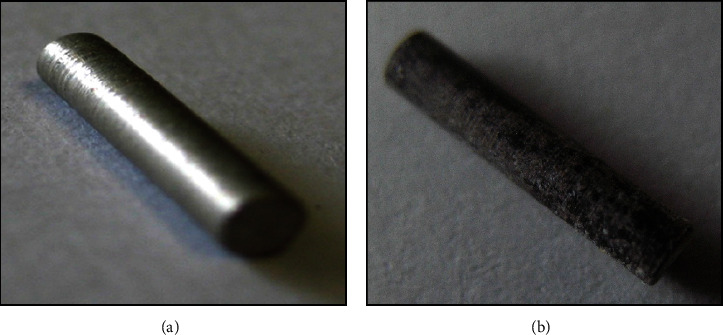
MgAg 1% sticks: before incubation (a); (b) after incubation.

The MgAg1% alloy was made from 99% magnesium and 1% silver with a weight of 10–70 mg, length of 1 cm, and a diameter of 0.2 cm (Institute of Materials Science, Leibniz University, Hannover, Germany) (Figure 1). These dimensions mentioned are for the original samples as received, and alloys which were used of samples with different dimensions and consequently different weight (10 to 70 mg) were cut from the original samples to be used in the different tests. Silver nitrate was purchased from Merck, Darmstadt, Germany. The MgAg1% sticks were incubated in 5 ml NaCl solution in a water bath for 15 days and the supernatant was used.

### 2.2. Determination of the Silver Concentrations in the Degradation Medium

**Figure 2:**
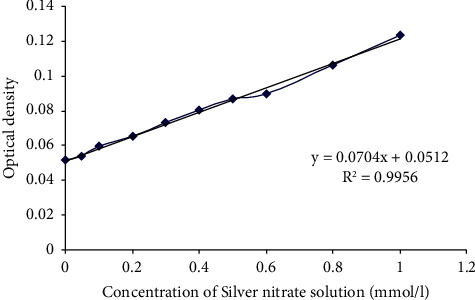
Calibration standard curve for the determination of silver concentrations, *n* = 6: calculated from the measured calibration function of the optical standards. Densities of total hardness measurement and the measurement of silver. *Y* = optical density; *X* = salt concentration; R^2^ = coefficient of determination (>0.95).

The amount of silver in the degradation medium of MgAg1% was measured using the kit NANOCOLOR Silver 3 test using a photometric procedure determination. Ag^+^ could be measured between 0.20 and 3.00 mg/l Ag^+^, as silver ions, react with an indicator to form a blue dye. In brief, for the determination of Ag^+^ concentrations in the degradation medium, the following was done:

A stock solution of 1 mmol/l AgNO_3_ was prepared, and a calibration standard curve of 0.05, 0.1, 0.2, 0.3, 0.4, 0.5, 0.6, and 0.8 mmol/l was prepared (Figure 2). Sterile MgAg 1% sticks were incubated in different volumes of the medium (1, 3, and 10 ml) or NaCl without adding any other supplements, in addition to one tube of the medium as the negative control. They were incubated in a water bath at 37°C with a shaking process for 25 days. The test was applied according to the manual manuscript with slight modifications. 28 *µ*l NANOCOLOR Silver 3 was added per well of a 96-well plate. 10 *µ*l of the NANOCOLOR Silver 3 R2 was added. Then, 80 *µ*l of the tested sample was added and all the reagents were shaken. Afterward, 10 *µ*l NANOCOLOR Silver 3 R3 was added per well, then it was mixed again and incubated for 10 min. The samples were in duplicate numbers. The measurements were performed on days 5, 10, 15, and 25 using a photometer with a wavelength of 620 nm.

### 2.3. Determination of the Magnesium and Calcium Concentrations in the Degradation Media

The Mg^2+^ and Ca concentrations in the degradation media were measured by the test kit NANOCOLOR hardness 20 using a photometric procedure. The test is based on measuring the total hardness of the medium, which is primarily maintained by Mg^2+^ and Ca but is also determined by barium and strontium ions. The color intensity depends on the total hardness of the surrounding medium. The calcium content can be obtained from the measured values of total hardness, and the Mg content can be calculated.

**Figure 3:**
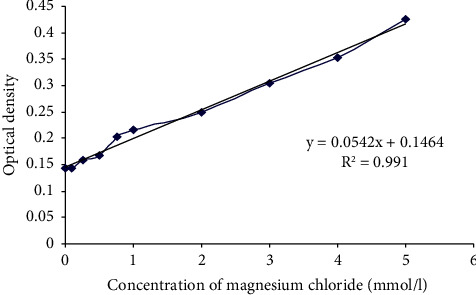
Calibration standard curve for the determination of magnesium concentrations, *n* = 6: calculated from the measured calibration function of the optical standards. Densities of total hardness measurement and the measurement of magnesium. *Y* = optical density; *X* = salt concentration; R^2^ = coefficient of determination (>0.95).

A stock solution of MgCl2 was prepared (10 mmol/l), and a calibrations standard curve of 0.1, 0.25, 0.5, 0.75, 1, 2, 3, 4, and 5 mmol/l was prepared (Figure 3). The samples were prepared from the degradation media by incubating MgAg1% sticks in different volumes of 10, 3, and 1 ml of medium or NaCl. One tube of pure medium or pure NaCl served as a negative control. Subsequently, 6 *µ*l of tested samples were added in each well of a 96-well plate, and then 180 *µ*l of NANOFIX hardness 20 R2 was added per sample and 6 *µ*l of hardness 20 R3 solution was added per sample. It was mixed by pipetting up and down. The samples were in duplicate numbers. The samples were measured at 570 nm.

Samples of higher concentrations above the calibration curve were diluted. The measurements were performed on days 5, 10, 15, and 25.

### 2.4. Detection of Antibacterial Activity

#### 2.4.1. Determination MgAg 1% Sticks on MIC and MBC of the Pathogenic Bacteria

The broth dilution method (31) used *Staphylococcus aureus*, and *Escherichia coli*, as indicator species to assess the MIC and MBC values of MgAg1% sticks.

- Different mass amounts of MgAg1% stick concentrations were incubated in 5 mg/ml to 0.156 mg/ml with adjusted bacterial concentration (10^8^ CFU/ml, 0.5 McFarland's standard) for 24 h at 37 °C. The MIC endpoint is the lowest concentration of MgAg1%, where no visible growth is seen in the tubes.
- One ml of pathogenic organism inoculum (2 × 10^5^ CFU/ml) was applied to tubes containing MgAg1% sticks growth media in a serial two-fold dilution.
- Turbidity tubes were measured after 24 hours as a growth indicator. The minimum inhibitory concentration (MIC) related to control is the lowest MgAg1% stick concentration that inhibits the organism's growth as detected by no visual turbidity.
- MBC (minimum bactericidal concentration) was described as the lowest concentration of test compounds that prevented visible bacterial growth on an LB agar plate after 24 hours of incubation at 37°C. The MBC was determined by testing the live microorganisms in the MIC test tubes that showed no growth. A loop of each of those tubes was inoculated on LB agar, and growth signs were observed. The presence of bacteria in the first tube is indicated by bacterial growth.

#### 2.4.2. Bouillon Dilution and Cultivation of Bacteria in Petri Dishes

Bouillon dilution test and cultivation of bacteria in Petri dishes were done by the Milchtierherden-Betreuungs-und Forschungsgesellschaft mbH, Bad Nenndorf, Germany (MBFG). The tests aim to determine the effect of Mg-Ag-NaCl solution on the growth of *E. coli* and *S. aureus*. In brief, MgAg1% sticks were incubated in 5 ml NaCl solution in a water bath for 15 days, and the degrading supernatants (MgAg1% sticks incubated in NaCl) were used for the bouillon dilution test and the cultivation of bacteria (*E. coli and S. aureus*). Firstly, the bouillon dilution test was done by preparing a bacterial suspension by incubating about 2 *E. coli* and *S. aureus* colonies (GK) in 100 ml buffered peptone water at 37°C for 24 hours on the shaker. The bacterial dilution series was 770 × 10^5^, 910 × 10^5^, 100 × 10^6^, and 210 × 10^6^ KbE/ml. Then, 2 test tubes were filled with 5 ml of bacterial suspension, then degrading was added to one of the tubes, and the other one serves as a negative control. Later, the number of bacterial colonies was determined. Secondly, *E. coli* and *S. aureus* colonies were cultivated in Petri dishes. Afterward, the bacterial colonies were incubated with the degrading supernatants, and the number of bacterial colonies was determined.

#### 2.4.3. Brilliant Black Reduction Test

The BRT was first described by KRAACK and TOLLE (1967) (15). This test is used for detecting antimicrobial agents in milk. The test medium consists of a mixture of nutrients, brilliant black indicator, bacteria *B. stearothermophilus var. calidolactis C953*, and other supplements. The results are visually interpreted by assessing the change in the color of the indicator present in the test medium. Penicillin *G* served as a positive control and milk as a negative control. MgAg1% sticks were incubated with 1, 3, and 10 ml of Dulbecco's MEM medium (DMEM), and the concentration of Mg^2+^ and Ag^+^ was noted in each volume. In addition, different concentrations of AgNO_3_ solution (control, 0.0001, 0.0003, 0.001, 0.003, 0.01, 0.03, 0.1, 0.3, and 1 mmol/l) were incubated with DMEM medium in test tubes. All tubes were incubated in a water bath for 5 days, then 100 *µ*l of each sample was added per well and incubated for 5-6 hours at 65°C.

### 2.5. Statistical Analysis

Data are presented as mean ± standard deviation (SD). Statistical analysis was carried out by two-way analysis of variance. Calculations were performed with GraphPad Prism® 5.03. *P* values < 0.05 were considered statistically significant.

## 3. Results

### 3.1. The Concentration of Magnesium and Silver

**Figure 4:**
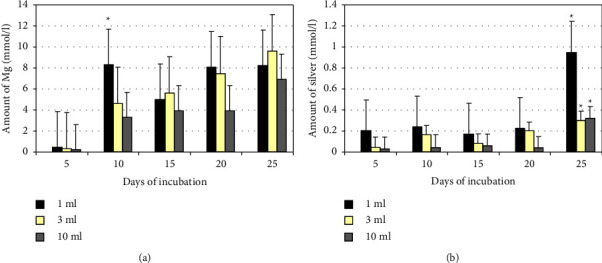
(a) Mean silver concentration (mmol/l) in degradation medium after incubation of MgAg1% sticks in 1, 3, or 10 ml medium. There is a significant increase of silver in degradation medium after 25 days of incubation, (^*∗*^*p* < 0.05, *n* = 6). (b) Mean silver concentration (mmol/l) in degradation medium after incubation of MgAg1% sticks in 1, 3, or 10 ml medium. There is a significant increase of silver in degradation medium after 25 days of incubation, (^*∗*^*p* < 0.05, *n* = 6).

The amount of magnesium and silver in MgAg1% degradation media with NaCl was about 0.8 mmol/l and 0.3 mmol/l after 15 days of incubation, respectively (Figure 4).

### 3.2. Determination of Minimum Inhibitory Concentration (MIC) and Minimum Bactericidal Concentration (MBC)

The MIC and MBC confirmed the qualitative antibacterial efficacy of the MgAg1% sticks, and the results are presented in Table 1. The minimum inhibitory concentration (MIC) of MgAg1% sticks was quantitatively investigated using the turbidity method. Results showed that a normal microbial growth inhibition zone was present. The antibacterial effect of various concentrations of MgAg1% relative to the tested pathogenic bacteria was highly significant. MIC and MBC ranged between 0.2 and 0.35 mL. Results show that the inhibition ratio of microbial strains increased to the maximum level of 620 nm added before reaching the limit, where MgAg1% inhibited growth. *S. aureus* obtained the highest inhibitory effect at concentrations of 0.2 mL and 0.35 mL on MIC and MBC.

### 3.3. Bouillon Dilution Test

**Figure 5:**
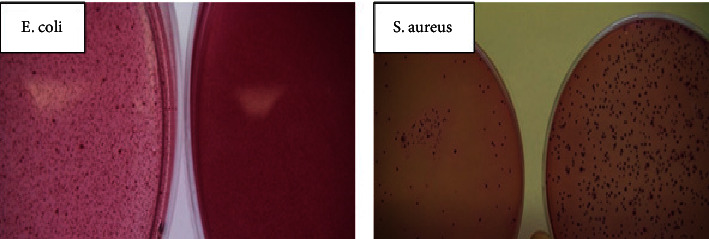
Bouillon dilution test: the number of the bacterial colonies of *E. coli* and *S. aureus* incubated with MgAg1% sticks were decreased 5 times compared to nonincubated ones.

The number of bacterial colonies of *E. coli* without incubation with MgAg1% stick was 1299, while after incubation with MgAg1% degradation medium after 15 days bacterial colonies decreased to 269. In addition, the number of bacterial colonies of *S. aureus* was 1194, while after incubation with MgAg1% degradation medium bacterial colonies decreased to 177. Thus, the MgAg1% sticks had an antibacterial activity with 5 times fewer colonies with bacterial dilutions 100 × 10^6^ and 210 × 10^6^ KbE/ml (Figure 5).

### 3.4. Brilliant Black Reduction (BRT-MRL Screening Test)

**Figure 6:**
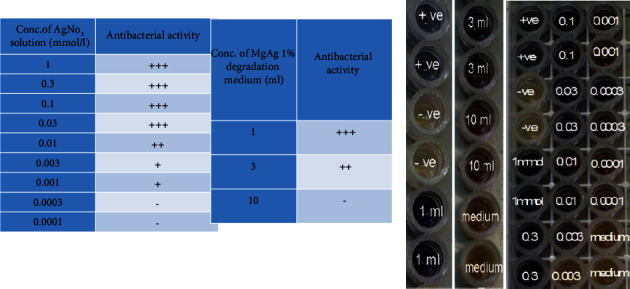
BRT screening test: + wells indicates antibacterial activity, while −ve wells indicate negative results. Positive results were seen in 1, 0.3, 0.1, 0.03, and 0.01 mmol/l of AgNO_3_ solution, 0.003 and 0.001 mmol/l of AgNO_3_ solution showed a suspicious result, while 0.0003 and 0.0001 mmol/l of AgNO_3_ solution showed a negative result. 1 ml and 3 ml of MgAg 1% degradation medium showed a positive result, and 10 ml of MgAg 1% degradation medium showed a negative result (^*∗*^*p* < 0.05, *n* = 6).

*B. stearothermophilus* bacteria shifted from brilliant black color to yellow or colorless, when treated with concentrations of AgNO_3_ equal to or below 0.03 mmol/L. While the color of the bacteria shifted to blue color at a volume higher than 3 ml of degradation medium of MgAg1% sticks. Positive results were seen in 0.03 up to 1 mmol/l of AgNO_3_ solution (Figure 6).

## 4. Discussion

In the present study, MgAg1% alloys are used for killing the pathogens responsible for causing mastitis. A combination of magnesium and silver was chosen due to its distinct properties and negligible side effects. Mg^2+^ serves as a biodegradable material, and Ag^+^ has a good antimicrobial effect. Depending on the concentration of Ag^+^ used, it can act as a bacteriostatic or bactericidal [10]. The use of antimicrobial therapy affects the human consumption of dairy products due to the presence of antibiotic residues in milk, it also increases labor and veterinary costs. As stated by Tilton, the soluble form of silver is most toxic to bacteria, therefore silver sticks are degraded in the solution of NaCl and used in the present study 7. Some clinical scenarios among humans that consume infected dairy products have public health importance, such as streptococcal sore throat, food poisoning due to *S. aureus*, tuberculosis, diphtheria, and brucellosis [11]. Antibiotic infusions into the mammary glands are the most common treatment method for mastitis [3, 12].

Results showed that the number of colonies of *E. coli* and *S. aureus* incubated with different volumes of MgAg1% in NaCl, decreased 5 times compared to the control. The concentration of silver which induced the antibacterial effect was 0.2–0.3 mmol/L which was measured after 15 days of incubation. Another study by Starodub and Trevors also showed the same result that *E. coli* strain *R*1 cultures treated with 0.3, 0.5, and 1 mM of AgNO_3_ reduced the final cell biomass [9]. Among the three concentrations used 0.5, and 1 mM showed a good antibacterial effect even after the incubation period was extended to 48 hours. The result of another study conducted by Kyun et al. showed a decrease in the bacterial count of *S. aureus* and *E. coli* due to silver ions [10].

The results obtained from the BRT test showed that *B. stearothermophilus* shifted from brilliant black color to yellow or colorless at a concentration equal to or more than 0.03 mmol/, which indicates that concentrations of AgNO_3_ solution starting from 1 up to 0.002 mmol/l have an antibacterial effect. Moreover, different volumes of degradation medium of MgAg1% shifted the blue color to yellow or colorless at volumes equal to or below 3 ml. The concentration of Ag^+^ in the solutions which showed an antibacterial effect was 0.02–1 mmol/l.

Jansen et al. and Schierholz et al. stated that the effect of Ag^+^ is continuous and long-lasting due to the oligodynamic effect of elementary Ag^+^ [8, 13]. Moreover, Bowswald et al. stated that the ability of many bacteria to produce a biofilm is reduced, and the likelihood of bacterial colonization is decreased when Ag^+^ is introduced [14]. Many studies stated the antibacterial activity of nonsteroidal anti-inflammatory drugs, local anaesthetics, phenothiazine such as chlorpromazine, levomepromazine, promethazine, trifluoperazine, methdilazine and thioridazine, antidepressants, antiplatelet, and statins [13]. Several studies have explored a possible protective effect of statins in decreasing the morbidity and mortality of many infectious diseases [15]. Various nonantibiotic agents exhibit antimicrobial activity via multiple and different mechanisms of action [14, 16]. The present study could be a useful aid for the treatment of mastitis. MgAg1% sticks can be useful as an alternative or adjunct to antibiotics for the treatment of mastitis at the dry-off period [17].

## 5. Conclusion

The current study was successful in treating the infections by the potential effect of the alloy sticks, and further investigations were done in reducing the culling rates in the dairy industry and minimizing the harmful effects on humans. However, it may be of interest to try to achieve higher concentrations in further investigations in vivo.

## Figures and Tables

**Table 1:** Effect of MgAg 1% sticks at different concentrations on the growth inhibition of *S. aureus* and *E. coli* using the turbidity method at 620 nm; values are means ±? standard deviation(SD)of at least three experiment(n?>3). Means with different letters within each coloum are significant at ?=0.01 level and means without letters are not significant N.S=not significant.

MgAg1% conc mL	*S. aureus*	*E. coli*
0	1.362a±0.012	1.360 a±0.051
0.05	1.324b±0.010	1.232b±0.002
0.1	1.224c±0.040	1.014c±0.022
0.15	1.000d±0.018	0.623d±0.060
0.2	0.729e±0.040	0.630e±0.026
0.3	0.610f±0.020	0.730f±0.020
0.35	0.360g±0.018	0.910g±0.016
0.5	0.176h±0.020	0.000h±0.000
1	0.000h±0.000	0.000h±0.000
LSD at ?=0.01	0.0492	0.0964

## Data Availability

All data are available and have been published in the thesis in https://elib.tiho-hannover.de/dissertations/nomiery_ws11.
